# Plasma Fibronectin Drives Macrophage Elongation via Integrin β3–Tie2 Axis in Blood Clots

**DOI:** 10.3390/cancers17233780

**Published:** 2025-11-26

**Authors:** Lynn M. Knowles, Hermann Eichler, Jan Pilch

**Affiliations:** 1 Institute of Clinical Hemostaseology and Transfusion Medicine, University Medical Center, Saarland University, 66421 Homburg, Germany; lynn.knowles@uks.eu (L.M.K.); hermann.eichler@uks.eu (H.E.); 2 Institute for Transfusion Medicine and Hemostasis, University Medical Center Augsburg, 86156 Augsburg, Germany

**Keywords:** fibrin, integrin β3, Tie2, M2 polarization

## Abstract

**Background/Objectives:** Plasma fibronectin (pFN) supports lung metastasis by promoting tumor cell invasion and survival in the context of blood clotting. Here, we set out to test if myeloid cells reiterate the clot-invasive mechanisms that have been established for tumor cells. **Methods:** We analyzed lung tissue sections from transgenic pFN-deficient mice for the co-localization of intravenously injected B16F1 tumor cells and the surrounding fibrin with myeloid cells, granulocytes, and macrophages. We also tested the role of pFN for macrophage differentiation and invasion in a three-dimensional fibrin matrix. **Results:** B16F1 melanoma cells, entrapped in the lungs of pFN-competent C57BL/6-*Fn(fl/fl)* *Mx-Cre^−^* mice, were surrounded by a fibrin matrix, CD11b-positive myeloid cells, and Gr-1-positive granulocytes within 1 h of intravenous injection, while homing F4/80-positive macrophages to lung-born tumor cells occurred within 16 h. Compared to pFN-competent C57BL/6-*Fn(fl/fl)* *Mx-Cre^−^* mice, the co-localization of CD11b^+^, Gr-1^+^, and F4/80^+^ cells with B16F1 cells was significantly reduced in the lungs of pFN-deficient C57BL/6-*Fn(fl/fl)* *Mx-Cre^−^* mice. Mechanistically, we found that fibrin–fibronectin complexes promoted macrophage adhesion, differentiation, and invasion in clotted plasma. The pro-invasive function of fibrin–fibronectin depended on the upregulation of integrin β3 and Tie2 expression in macrophages and was reversed after knocking-down integrin β3 and Tie2 with siRNA. **Conclusions:** Our results suggest that blood clotting plays an important role in the recruitment of macrophages to circulating tumor cells and that the underlying mechanism of macrophage recruitment involves fibrin–fibronectin complexes, integrin β3, and Tie2.

## Simple Summary

This article investigates the role of the adhesion protein plasma fibronectin (pFN) for homing of white blood cells to the metastatic niche of lungs. The study demonstrates that myeloid cells, a subset of white blood cells, colocalize with tumor cells and blood clot in the lung vasculature of mice and that this process depends on the availability of pFN. On a functional level, our data show that the presence of pFN in clot contributes to the differentiation of monocytes into clot-invasive M2 macrophages that have been shown to enhance tumor function.

## 1. Introduction

Metastasis is a lethal complication of cancer that involves the systemic dissemination of tumor cells through the blood flow and subsequent growth in distant organs. In order to metastasize, tumor cells are equipped with a specific set of properties including the capacity to escape from the primary tumor, extravasate in the target organ, and proliferate [1]. At the same time, tumor cells need to escape from the immune response, which consists of natural killer cells, dendritic cells, and effector T cells [2]. The main escape mechanism is called immuno-editing, which is a selective process that causes clones of the least immunogenic tumor cells to survive. However, immunosuppression can also be achieved by a tumor microenvironment that typically exhibits hypoxia, low pH, and high levels of reactive oxygen species [3]. In addition, tumor cells actively turn a hostile microenvironment into friendly territory by secreting cytokines and chemokines that mediate the recruitment of immunosuppressive myeloid cells, regulatory dendritic cells, and regulatory T cells [2].

The majority of immune cells within the tumor tissue are myeloid cells, and chief among these are tumor-associated macrophages (TAMs) [4]. TAMs can be roughly divided into classically (M1) and alternatively (M2) activated macrophages, with the former being more prevalent in early-stage tumors, while the latter are typically found in later stages [5]. M1 macrophages are thought to be anti-tumorigenic, as they express high levels of TNFα, iNOS, and reactive oxygen species; however, M1 macrophages have also been shown to promote neoplastic transformation and metastasis based on the secretion of these factors [6]. M2 macrophages are tumor-supporting, as they secrete factors that promote immune escape, tumor growth, and angiogenesis [7,8]. In line with this, it has been shown that the extent of macrophage infiltration in tumors correlates positively with poor outcomes [9]. While M2 macrophages support angiogenesis, this function is particularly pronounced in Tie2-expressing macrophages (TEMs), which are similar to M2 macrophages but constitute an independent TAM subset [10].

The blood clotting system constitutes an important immune escape mechanism for blood-borne tumor cells, which co-opt fibrin(ogen) and activated platelets for metastasis to the lung [11]. However, the protective function of fibrin–platelet clots against natural killer cells appears to be an indirect effect that results from the pro-invasive properties of clotted plasma, thereby supporting the escape from natural killer cells through expedited tumor cell extravasation [12]. This mechanism depends, in a certain subset of tumor cells, on the presence of plasma fibronectin (pFN), an abundant adhesion protein that circulates in the blood stream and supports lung metastasis through promoting tumor cell invasion, survival, and colonization [13,14]. Notably, the tumor-promoting effects of pFN were contingent on coagulation factor XIIIa-mediated crosslinking of pFN to fibrin as the last step of the clotting cascade. This process resulted in the formation of fibrin–fibronectin complexes that were able to induce activation of the integrin receptor αvβ3 and subsequent upregulation of the receptor tyrosine kinase Tie2.

It has been shown that macrophage homing to tumors is impaired in transgenic mice with deficiencies in fibrin(ogen) and integrin β3, suggesting that adhesive interactions with blood clots are relevant for macrophage recruitment to tumor cells and tissues [15,16]. Depending on the tissue context, adhesive interactions of macrophages with clots can either induce inflammation or promote regeneration [17,18]. However, research on macrophages from patients with severe clotting deficits indicate that adhesive interactions with clotted plasma induce a regenerative M2 macrophage phenotype by default and that the default polarization of macrophages into an M2 phenotype is impaired in patients with clotting impairments [19]. Previous data suggest that pFN exerts critical functions as a component of fibrin–fibronectin complexes in clotted plasma [14]. Here, we tested the role of fibrin–fibronectin complexes for the recruitment of macrophages to lung metastatic tumor cells in vivo and for the differentiation of monocytes into M2 macrophages in vitro.

## 2. Materials and Methods

### 2.1. Metastasis Model

Transgenic C57BL/6-*Fn(fl/fl)* *Mx-Cre^+^* mice become pFN-deficient by postnatally deleting the fibronectin gene in the liver using polyinosinic/polycytidylic acid (Sigma-Aldrich, Saint Louis, MO, USA), whereas C57BL/6-*Fn(fl/fl)* *Mx-Cre^−^* mice maintain the capacity to generate pFN after treatment with polyinosinic/polycytidylic acid and, therefore, served as controls. To analyze co-localization with myeloid cells, B16F1 melanoma cells (American Type Culture Collection, ATCC, Manassas, VA, USA) were labeled with Cytotracker™ (Thermo Fisher Scientific, Waltham, MA, USA) and injected at 5 × 10^5^ alternatingly into the tail vein of transgenic pFN-deficient *Fn(fl/fl)* *Mx-Cre^+^* mice and their *Mx-Cre^−^* littermates (*n* = 8–12). Both 1 and 16 h after tumor cell injection, lung tissue was harvested, snap frozen in OCT, and the blocks were cut at 10 µm for staining. Lung sections were fixed with 4% paraformaldehyde and incubated with biotinylated mouse fibrin(ogen) antiserum (Nordic MUbio, Susteren, The Netherlands), anti-CD11b (Bio-Rad, Hercules, CA, USA, clone M1/70.15), anti-Gr-1 (BD Biosciences, San Jose, USA, clone RB6-8C5), or F4/80 (eBioscience, San Diego, CA, USA, clone BM8), followed by incubation with Alexa Fluor 594-conjugated secondary antibody or Alexa Fluor 647-streptavidin (Thermo Fisher Scientific). To visualize nuclei, cells were stained with mounting media containing DAPI (Vector Laboratories, Newark, NJ, USA). Stained lung sections were analyzed for the co-localization of fibrin, CD11b^+^ myeloid cells, Gr-1^+^ neutrophils, F4/80^+^ macrophages, and tumor cells using a fluorescence microscope (Zeiss Axioplan 2, Carl Zeiss Meditec AG, Oberkochen, Germany). Images were processed with Adobe Photoshop (Adobe, San Jose, CA, USA). To quantify co-localization, each optical field was scored for the total number of tumor cells and the number of CD11b^+^, Gr-1^+^, or F4/80^+^ cells adjacent to any given tumor cell within the optical field. The ratio between the number of tumor cell-adjacent CD11b^+^, Gr-1^+^, or F4/80^+^ cells and the total number of tumor cells is shown.

### 2.2. 3-Dimensional Cell Culture

Peripheral blood mononuclear cells (PBMCs) were isolated from blood using Ficoll Paque Premium according to the manufacturer’s instructions (GE Healthcare, Chicago, IL, USA). Monocytes were separated from the PBMC population using the Dynabeads^®^ Untouched™ Human Monocytes Kit (Thermo Fisher Scientific) and cultured in RPMI media supplemented with 10% FBS and L-glutamine at 37 °C under a humidified 5% CO_2_ atmosphere. THP-1 human monocytic, 786-0 kidney cancer, and U87MG glioblastoma cells were purchased from ATCC and were cultured according to the manufacturers’ specifications. THP-1 cells were maintained in RPMI media containing 10% FBS, L-glutamine, and 0.05 mM beta mercaptoethanol. 786-0 cells were cultured in DMEM media containing 10% FBS, and U87MG cells were grown in minimal essential medium supplemented with 10% FBS, L-glutamine, minimal essential medium vitamins, nonessential amino acids, and sodium pyruvate. All cell culture reagents were purchased from Gibco (Thermo Fisher Scientific). For the 3-dimensional (3D) culture, cells were mixed with 2 mg/mL fibrinogen (Enzyme Research Laboratories, Inc., South Bend, IN, USA) in the presence of 2 mM CaCl_2_ and 25 µg/mL FXIII (Enzyme Research Laboratories) to generate fibrin clots, as previously described [13]. For fibrin–fibronectin clots, 200 µg/mL pFN (Sigma-Aldrich) was added to the mixture. For clotted plasma, cells were mixed with human plasma (US Biological, Salem, MA, USA). In all cases, clotting was induced with 2.5 U/mL thrombin (Sigma-Aldrich), and 15 µL suspensions were pipetted onto tissue culture plates and inverted at room temperature for 10 min to solidify. Embedded cells were incubated with media supplemented with FBS at 37 °C under a humidified 5% CO_2_ atmosphere. PBMC-derived monocytes were treated with 125 ng/mL granulocyte–macrophage colony-stimulating factor (GM-CSF; Cellgenix, Freiburg, Germany) to generate M1 macrophages or 100 ng/mL macrophage colony-stimulating factor (M-CSF; Miltenyl Biotec, Teterow, Germany) to generate M2 macrophages. After 7 days, M-CSF-differentiated M2 macrophages were further activated with 20 ng/mL interleukin 4 (IL-4; Cellgenix) to further enhance their M2 characteristics or activated with 1 µg/mL interferon-gamma (IFN-γ; Thermo Fisher Scientific) and 1 µg/mL lipopolysaccharide (LPS; Sigma-Aldrich) to promote M1 polarization [20]. To induce macrophage differentiation in THP-1 cells, media was supplemented with 160 nM phorbol myristate acetate (PMA; Sigma-Aldrich) [21].

### 2.3. Analysis of Cell Morphology

Cells in the 3D culture were analyzed at designated times by bright field microscopy using a Nikon Eclipse TS100 (Nikon, Tokyo, Japan) or Zeiss Primo Vert microscope (Carl Zeiss Meditec AG), as previously described [12]. In brief, cells were counted based on their shape in an average of 4 distinct optical fields per clot in at least 3 clots. Macrophages or tumor cells with a spread, extended phenotype were classified as elongated, while circular cells were counted as round. Results show the number of elongated cells as a % of the total cell number per microscopy field. To assess the actin cytoskeleton, macrophages were grown on glass culture slides (Corning Inc., Corning, NY, USA), fixed with 4% paraformaldehyde, permeabilized with 0.2% triton X-100, and stained with the macrophage marker CD68 (Abcam, Cambridge, United Kingdom) or isotype control, followed by incubation with Alexa Fluor 488-conjugated secondary antibody (Thermo Fisher Scientific) and Alexa Fluor 546-phalloidin (Thermo Fisher Scientific). Nuclei were stained with mounting media containing DAPI (Vector Laboratories). Fluorescence was visualized on a Nikon Eclipse Ni fluorescent microscope (Nikon) equipped with NIS elements BR 4.2 software.

### 2.4. Gene Silencing

THP-1, 786-0, or U87MG were grown for 24 h prior to transfection with 25 nM fibronectin (Dharmacon On-TARGETplus SMARTpool L-009853-00, Horizon Discovery, Cambridge, United Kingdom), integrin β3 (Dharmacon On-TARGETplus SMARTpool L-004124-00), Tie2 (Dharmacon On-TARGETplus SMARTpool L-003178-00), or a non-targeting control (Dharmacon On*-*TARGETplus D-001810-10) siRNA. SMARTpool siRNA contains a pool of four siRNA sequences directed against the target gene. Cells were transfected in Opti-MEM medium (Thermo Fisher Scientific) using Lipofectamine 2000 reagent (Thermo Fisher Scientific) according to manufacturer’s instructions. After 6 h, cells were placed in normal culture medium and grown for an additional 42 h. Target knockdown was confirmed by Western blot or real-time PCR analysis.

### 2.5. Real-Time PCR

THP-1 cells were monitored for changes in gene expression 48 h after treatment with or without PMA. Total RNA was isolated from cells using the Qiagen RNeasy kit (Qiagen, Venlo, The Netherlands). RNA expression was measured using primers targeting *TEK* (Hs00945146_m1), *ITGB3* (Hs01001469_m1), and TaqMan Gene Expression assay reagents (Thermo Fisher Scientific). RT-PCR was performed on an Applied Biosystems StepOnePlus™ Real-Time PCR System (Thermo Fisher Scientific). Values were normalized to *TBP* (Hs00427620_m1) as a reference gene.

### 2.6. Cell Adhesion Assay

Forty-eight well non-tissue culture treated plates (Corning Inc.) were coated with 10 µg/mL fibrin(ogen) or bovine serum albumin (BSA) overnight at 4 °C and then blocked with 1% BSA for 1 h at 37 °C. PMA-differentiated THP-1 cells (2 × 10^5^) were suspended in HEPES-Tyrode’s buffer containing 0.1% BSA and 2 mM CaCl_2_ and added to the culture plates in the presence of 100 µg/mL pFN or solubilized clot material from plasma (±pFN), fibrin–fibronectin, or fibrin prepared as previously described [14]. Cells were allowed to attach for 1 h at 37 °C. Plates were washed to remove non-adherent cells, and attached cells were lysed with para-nitrophenol phosphate (5 mg/mL in 50 mM sodium acetate, 1% Triton X-100, pH 5.2) for 30 min at 37 °C. Reactions were stopped by the addition of 0.3 M sodium hydroxide and the absorption was measured at 405 nm on a plate reader (Tecan SPARK, Tecan, Männedorf, Switzerland).

### 2.7. Statistical Analysis

Significance was determined using Student’s two-tailed t-test or a one-way ANOVA followed by post hoc Tukey’s multiple comparisons test (Prism 5, GraphPad Software, Boston, MA, USA). Treatment differences with a two-sided *p* value < 0.05 were considered significantly different. Error bars show mean ± SEM.

## 3. Results

### 3.1. Homing of Myeloid Cells to Clot-Associated Tumor Cells Is Reduced in pFN-Deficient Mice

**Figure 1:**
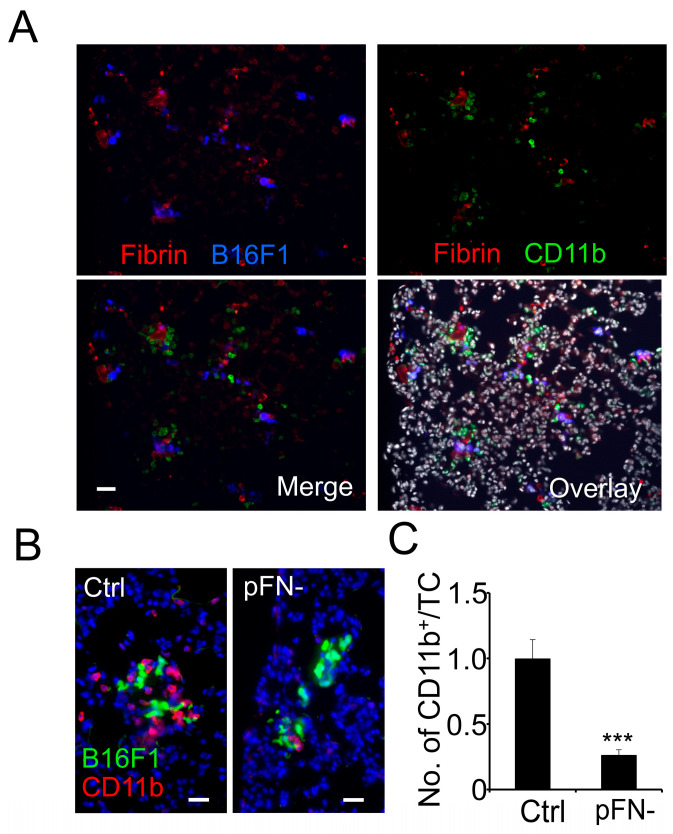
Myeloid cell homing to clot-associated tumor cells is reduced in pFN-deficient mice. (**A**) Lung tissue from pFN-competent C57BL/6-*Fn(fl/fl)* *Mx-Cre^−^* mice isolated 1 h after injection with fluorescent B16F1 cells (blue) was stained for fibrin (red) and the myeloid cell marker CD11b (green). Fibrin-rich areas are shown surrounded by CD11b^+^ myeloid cells and B16F1 cells (Merge). DAPI-stained nuclei are shown in gray in the Overlay. (**B**,**C**) Co-localization of B16F1 cells (green) with CD11b^+^ myeloid cells (red) in lungs from pFN-competent C57BL/6-*Fn(fl/fl)* *Mx-Cre^−^* (**B**; Ctrl, **left panel**) and pFN-deficient C57BL/6-*Fn(fl/fl)* *Mx-Cre^+^* mice (**B**; pFN-, **right panel**) 1 h after tumor cell (TC) injection is shown as the ratio between the number of tumor cell-adjacent CD11b^+^ and the total number of TCs per optical field (**C**; *n* = 12). Nuclei are stained with DAPI (blue). *** *p* < 0.001. Scale bar, 50 µm.

Our previous research showed that pFN promotes experimental lung metastasis in vivo and tumor cell invasion in clots in vitro [14]. Here, we tested if pFN mediates the homing of inflammatory cells to metastatic tumor cells arrested within blood clots in the lungs. To this end, B16F1 melanoma cells were injected into the tail vein of transgenic C57BL/6-*Fn(fl/fl)* Mx-Cre^+^ mice, which become pFN-deficient upon postnatal deletion of the FN gene in the liver. pFN-competent C57BL/6-*Fn(fl/fl)* Mx-Cre^−^ littermates served as controls. One hour after the injection of fluorescent-labeled B16F1 cells, lung tissue was harvested, snap frozen in OCT, and stained for the myeloid cell marker CD11b as well as fibrin(ogen). Subsequent fluorescence microscopy analysis of lung tissue sections from pFN-competent C57BL/6-*Fn(fl/fl)* Mx-Cre^−^ mice revealed extensive fibrin formation around tumor cells (Figure 1A). Moreover, fibrin-associated tumor cells were surrounded by large numbers of CD11b^+^ myeloid cells, which were in direct contact with the fibrin clot rather than with the tumor cells themselves. While recruitment of CD11b^+^ cells to metastatic tumor cells was extensive in pFN-competent C57BL/6-*Fn(fl/fl)* Mx-Cre^−^ mice, we detected significantly less CD11b^+^ cells adjacent to tumor cells in lungs from pFN-deficient C57BL/6-*Fn(fl/fl)* Mx-Cre^+^ mice (Figure 1B,C). Together, these results suggest that pFN plays an important role in the initial recruitment of CD11b^+^ myeloid cells to metastatic tumor cells in mice in vivo.

### 3.2. Association of Granulocytes and Macrophages with Metastatic Tumor Cells Is Reduced in pFN-Deficient Mice

**Figure 2:**
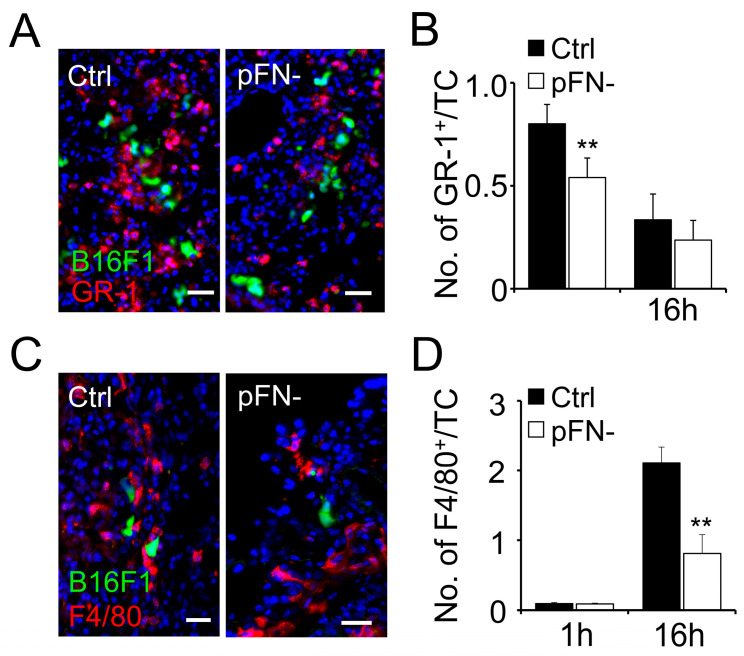
Granulocyte and macrophage co-localization with metastatic tumor cells is reduced in pFN-deficient mice. (**A**,**C**) Co-localization of B16F1 cells (green) with GR-1^+^ granulocyte cells (**A**; red) or F4/80^+^ macrophages (**C**; red) is shown in representative micrographs of lungs from pFN-competent C57BL/6-*Fn(fl/fl)* *Mx-Cre^−^* (Ctrl, **left panel**) and pFN-deficient C57BL/6-*Fn(fl/fl)* *Mx-Cre^+^* mice (pFN-, **right panel**) isolated 1 h (**A**) or 16 h (**C**) after tumor cell (TC) injection. (**B**,**D**) The ratio between the number of tumor cell-adjacent GR-1^+^ (**B**) or F4/80^+^ (**D**) cells and the total number of TCs per optic field were scored after 1 h (*n* = 12) and 16 h (*n* = 8). Nuclei are stained with DAPI (blue). Scale bar, 50 µm. ** *p* < 0.05.

To further dissect the inflammatory reaction induced by circulating tumor cells, we performed immunohistochemistry of murine lung tissues harvested 1 and 16 h after intravenous tumor cell injection for the granulocyte marker Gr-1 and the macrophage marker F4/80. Subsequent fluorescence microscopy analysis revealed that the homing of Gr-1^+^ granulocytes to metastatic B16F1 tumor cells was significantly lower in the lungs of pFN-deficient C57BL/6-*Fn(fl/fl)* *Mx-Cre^+^* mice 1 h after tumor cell injection compared to pFN-competent C57BL/6-*Fn(fl/fl)* *Mx-Cre^−^* mice (Figure 2A,B). At 16 h after tumor cell injection, the reduction in tumor cell-associated granulocytes in the lungs of pFN-deficient C57BL/6-*Fn(fl/fl)* *Mx-Cre^+^* mice was still detectable but no longer significant. The association of F4/80^+^ macrophages with B16F1 tumor cells, on the other hand, was negligible in lungs from pFN-competent C57BL/6-*Fn(fl/fl)* *Mx-Cre^−^* as well as pFN-deficient C57BL/6-*Fn(fl/fl)* *Mx-Cre^+^* mice harvested 1 h after tumor cell injection but increased considerably after 16 h (Figure 2C,D). Notably, the increase in tumor cell-associated macrophages after 16 h depended on the presence of pFN, as we detected significantly more macrophages in direct vicinity of tumor cells in the lung tissue of pFN-competent C57BL/6-*Fn(fl/fl)* *Mx-Cre^−^* mice than in the lungs of pFN-deficient C57BL/6-*Fn(fl/fl)* *Mx-Cre^+^* mice. Therefore, our data indicate that pFN supports the initial recruitment of granulocytes as well as macrophages homing to tumor cells later on.

### 3.3. M2-Polarized Macrophages Invade Clotted Plasma In Vitro

**Figure 3:**
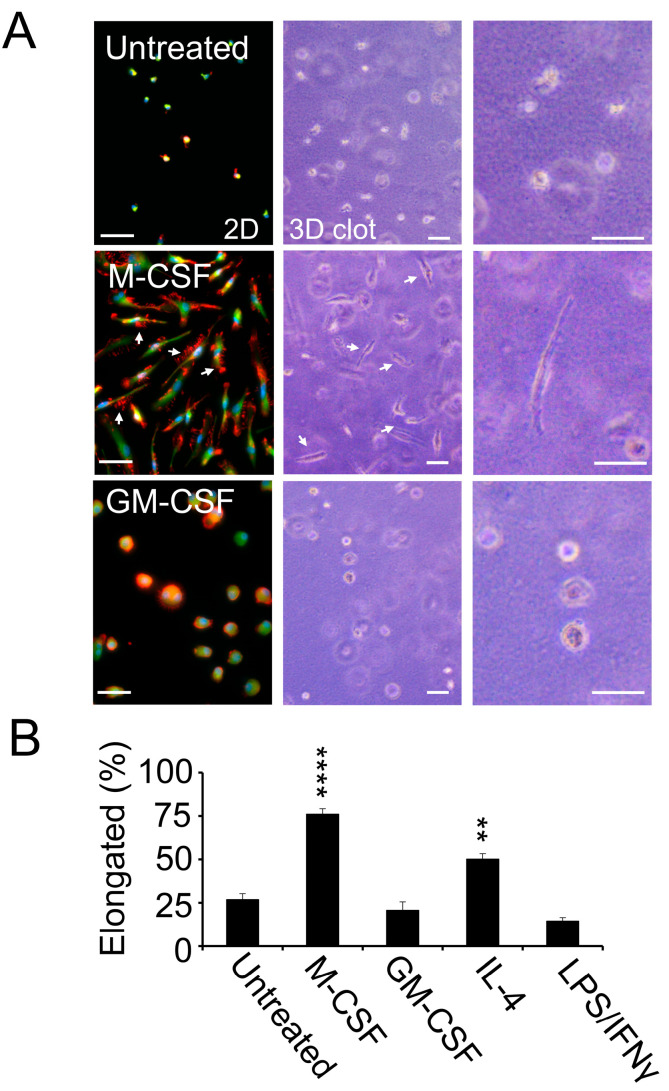
M2-polarized macrophages invade clotted plasma in vitro. (**A**) Representative fluorescence microscopy and phase contrast images of primary human macrophages from healthy donors after treatment for 8 days with vehicle control (untreated; **top**), M-CSF (**middle**), or GM-CSF (**bottom**). Fluorescence microscopy (**left panel**) shows merged images of macrophages cultured on cell culture slides stained for the macrophage marker CD68 (green), F-actin (red), and nuclei (blue). Phase contrast microscopy (**middle panel**, low magnification; **right panel**, high magnification) shows macrophages cultured in a 3D matrix of clotted plasma. White arrows denote filopodia (**left panel**) and elongation (**left** and **middle panels**) of M-CSF treated macrophages. Scale bar, 50 µm. (**B**) Phase contrast microscopy images of plasma clots were analyzed for percent elongated macrophages per optical field after treatment with vehicle control (untreated), M-CSF, GM-CSF, or following the addition of IL-4 or the combined addition of LPS and IFNγ for 24 h to the M-CSF treated cultures (*n* = 9). ** *p* < 0.01, **** *p* < 0.0001.

Given the increase in tumor cell-associated macrophages in the lungs of pFN-competent mice over time, we decided to focus on the effect of pFN on macrophage differentiation and invasion in the context of clotting. Since pFN becomes associated with fibrin during plasma clotting, we embedded freshly isolated monocytes from the peripheral blood of healthy blood donors in a 3D matrix of clotted plasma in vitro and added M-CSF to induce the differentiation of monocytes into M2-polarized macrophages [19]. In monocytes grown on culture slides, M-CSF treatment is accompanied by a typical change in cell shape from round to elongated and the formation of F-actin-rich filopodia (Figure 3A). The M-CSF-induced shape change in M2-polarized macrophages from round to elongated was reiterated in monocytes embedded in 3D clotted plasma after 8 days of M-CSF treatment alone or in combination with interleukin-4 to reinforce M-CSF-induced M2 polarization (Figure 3A,B and Figure S1). Monocytes that were treated with GM-CSF to induce M1 polarization stayed round during macrophage differentiation and did not undergo a relevant shape change, whether they were attached to culture slides or embedded in 3D clotted plasma (Figure 3A,B and Figure S1). M1 macrophages could also be generated by adding LPS and IFN-γ to M2 macrophages embedded in clotted plasma, which changed their phenotype from elongated to round within 24 h of the pro-inflammatory treatment (Figure 3B and Figure S1). Our data, therefore, demonstrate that the typical shape change in M2 macrophages from round to elongated takes place after embedding monocytes in a 3D matrix of clotted plasma in the presence of M-CSF with or without IL-4, while inflammatory macrophages take on a round shape. Since it is generally accepted that a change in cell shape from round to elongated in a 3D matrix involves cell-invasive properties, we considered the elongated M2 macrophages surrounded by clotted plasma to be clot-invasive [22].

### 3.4. pFN Supports a Pro-Invasive M2 Macrophage Phenotype

**Figure 4:**
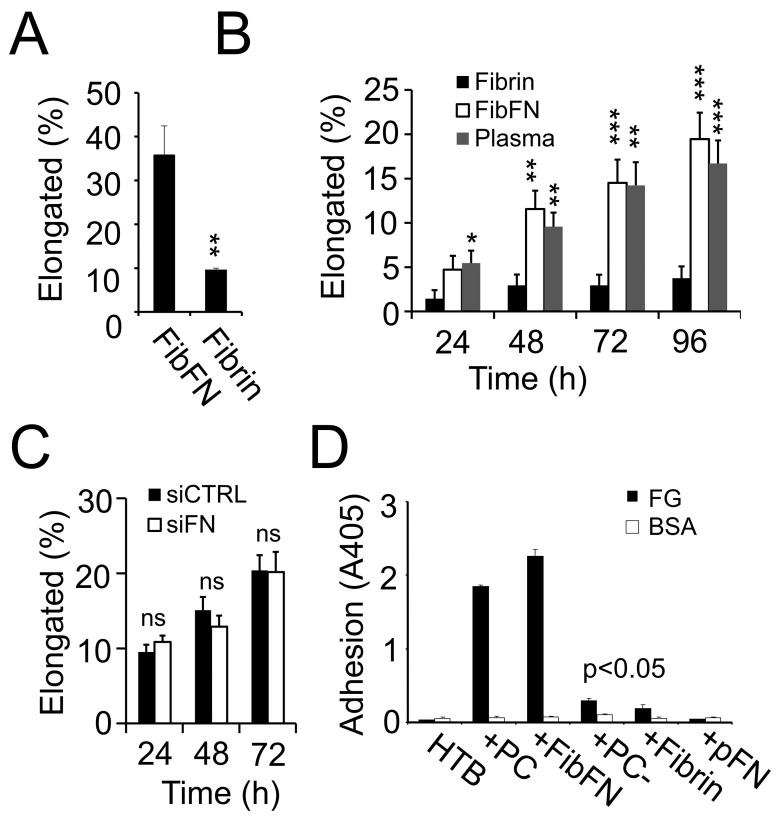
pFN supports a pro-invasive M2 macrophage phenotype. (**A**) M-CSF-treated primary human macrophages were embedded in fibrin or fibrin–fibronectin (FibFN) clots for 7 days and analyzed for the number of elongated macrophages per optic field as a percent of total cell count (*n* = 12). (**B**) Percent elongated PMA-treated THP-1 macrophages in fibrin, FibFN, or clotted plasma over time (*n* = 9). (**C**) Percent elongated PMA-treated THP-1 macrophages in FibFN after treatment with siRNA targeting FN or non-targeting control over time (*n* = 6). (**D**) PMA-treated THP-1 cells were suspended in HEPES-Tyrodes buffer alone or in the presence of pFN or solubilized clot material from plasma ± pFN (PC, PC-), fibrin–fibronectin (FibFN), or fibrin and examined for adhesion to fibrinogen (FG)- or bovine serum albumin (BSA)-coated plates (*n* = 4).* *p* < 0.05, ** *p* < 0.01, *** *p* < 0.001, ns, non-significant.

pFN is an integral component of clotted plasma as it becomes cross-linked to fibrin through the enzymatic activity of activated coagulation factor XIII [23]. To determine if the M-CSF-mediated shape change in M2 macrophages depends on pFN, we embedded monocytes isolated from the peripheral blood of healthy blood donors in a 3D matrix of fibrin–fibronectin, which we generated by mixing fibrinogen with pFN, thrombin, and calcium. A 7-day culture course in 3D fibrin–fibronectin in the presence of M-CSF resulted in the formation of elongated invasive macrophages in almost half of the embedded monocytes, while only a small fraction of monocytes elongated after embedding in a 3D matrix of pure fibrin without the addition of pFN (Figure 4A). Reproducing the data derived from primary human macrophages, we detected significantly more elongated THP-1 macrophages after embedding in 3D clotted plasma and fibrin–fibronectin than in 3D fibrin, which failed to support the shape change in macrophages completely (Figure 4B). The dependency of macrophages on pFN aligns with our findings that macrophages are unable to establish a fibronectin matrix on their own and that knocking down fibronectin with siRNA in THP-1 macrophages did not reverse cell elongation induced by fibrin–fibronectin (Figure 4C and Figure S2). The pro-invasive function of pFN depended on complex formation with fibrin because only solubilized fibrin–fibronectin complexes were able to stimulate the attachment of THP-1 cells to fibrinogen (Figure 4D). pFN, fibrin, or pFN-depleted plasma clot fragments, on the other hand, were ineffective. Together, these results indicate that fibrin–fibronectin complexes in blood clots stimulate adhesive interactions in macrophages that in turn support macrophage invasion and M2 polarization.

### 3.5. Macrophage Polarization and Invasion in Fibrin–Fibronectin Is Supported by Integrin αvβ3 and Tie2

**Figure 5:**
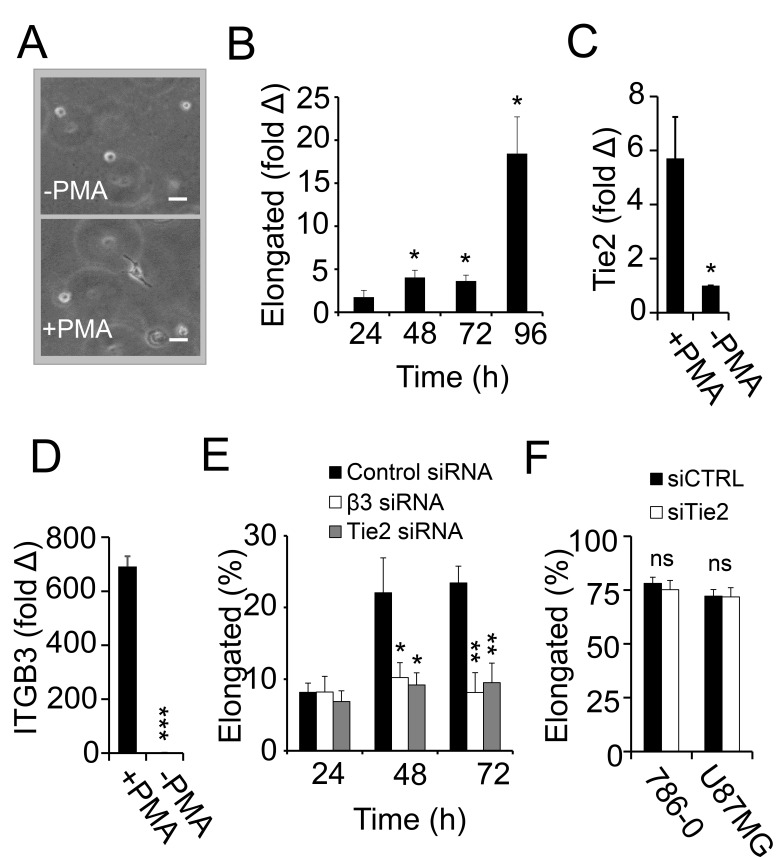
Tie2 promotes pFN-dependent macrophage elongation. (**A**) Representative images of THP-1 cells embedded in a 3D matrix of fibrin–fibronectin in the presence (+PMA) or absence of PMA (-PMA). Scale bar, 20 µm. (**B**) Percent elongated THP-1 cells in fibrin–fibronectin per optical field after treatment with PMA over time. Values are shown as fold change over -PMA at each time point (*n* = 6). (**C**,**D**) *TEK*/Tie2 (**C**) and *ITGB3* (**D**) mRNA expression by RT-PCR in THP-1 macrophages after treatment with PMA for 48 h compared to THP-1 monocytes without PMA normalized to *TBP* (*n* = 3). *TEK*/Tie2 and *ITGB3* expression in THP-1 monocytes without PMA is set to 1. (**E**) Percent elongated PMA-treated THP-1 macrophages in FibFN after treatment with siRNA targeting integrin β3, Tie2, or non-targeting control over time (*n* = 6). (**F**) 786-0 or U87MG tumor cells display shape change and elongation in fibrin–fibronectin 48 h after transfection with Tie2 siRNA or non-targeting control (*n* = 6). * *p* < 0.05, ** *p* < 0.01, *** *p* < 0.001, ns, non-significant.

We previously demonstrated that fibrin–fibronectin promotes integrin αvβ3 activation on THP-1 cells and that this mechanism correlates with Tie2-mediated invasion of B16F1 melanoma cells in fibrin–fibronectin [13,14]. To probe the adhesive mechanism governing macrophage elongation in fibrin–fibronectin, we tested the role of macrophage differentiation and found that untreated, monocytic THP-1 cells spread poorly in fibrin–fibronectin compared to PMA-induced macrophages (Figure 5A,B). Next, we analyzed the expression levels of integrin αvβ3 as well as the receptor tyrosine kinase Tie2, which were both upregulated in PMA-treated macrophages compared to untreated-naive monocytes (Figure 5C,D). The increase in integrin β3 and Tie2 expression was functionally relevant, as transfection with siRNA against integrin β3 and Tie2 caused a significant decrease in THP-1 cell elongation and invasion in 3D fibrin–fibronectin compared to transfection with non-targeted siRNA (Figure 5E and Figure S3). While the Tie2 knockdown effectively inhibited elongation of THP-1 macrophages, it had no effect on tumor cells such as 786-0 and U87MG, which generate elongated invadopodia in fibrin independent of pFN (Figure 5F) [13,24]. Together, our results suggest that macrophage differentiation supports shape change and invasion in fibrin–fibronectin by increasing the expression of genes involved in pFN-interacting pathways such as integrin αvβ3 and Tie2. Moreover, while integrin αvβ3 is also relevant for invasion in fibrin, they suggest that Tie2 represents a fibrin–fibronectin-specific pathway.

## 4. Discussion

Clotting induced by tumor cells serves an important purpose in metastasis, as it promotes tumor cell extravasation in the lung vasculature [12,14,25]. We show here that clotting coincides with the homing of myeloid cells to metastatic tumor cells. This process is supported by pFN, which enhances the recruitment of granulocytes and macrophages to tumor cells entrapped in the lungs. Mechanistically, we found that the association of pFN with fibrin promotes the differentiation of monocytes into M2 macrophages. Macrophage differentiation in fibrin–fibronectin clots encompasses adhesive and invasive properties that depend on the presence of pFN in clots and are reversed after knocking down integrin β3 and Tie2 with siRNA. Together, these data suggest that pFN connects tumor metastasis to wound healing and inflammation in the context of clotting.

Myeloid cells have been shown to support tumor cell metastasis as they generate an immunosuppressive microenvironment, promote vascular permeability, and support angiogenesis [26]. Characteristic for myeloid cells is the expression of integrin αMβ2 (i.e., CD11b), an adhesion receptor that promotes monocyte and granulocyte infiltration through interactions with fibrinogen or fibrin [27]. Accordingly, we saw close interaction between clots that formed around intravenously injected tumor cells entrapped in lungs of immunocompetent mice and cells positive for the myeloid marker CD11b. Subsequent experiments demonstrated that the expression of the fibrin(ogen) receptor integrin αMβ2 on myeloid cells alone was not sufficient to promote homing to metastatic tumor cells in the lungs of pFN-deficient C57BL/6-*Fn(fl/fl)* *Mx-Cre^+^* mice, even though pFN-deficient mice exhibit no obvious clotting defects and generate clots around metastatic tumor cells to the same extent as pFN-competent wildtype mice [14,28]. While delayed macrophage homing in transgenic mice with fibrin(ogen) or coagulation factor IX deficiency correlates with impaired clot formation, we hypothesize here that the lack of pFN affects the adhesive function of myeloid cells with clotted plasma rather than clot formation itself [29,30]. The finding that pFN can support the recruitment of myeloid cells was confirmed by showing a reduced co-localization of subsets of myeloid cells such as granulocytes and macrophages with tumor cells in the lungs of pFN-deficient mice. These experiments also confirmed the typical sequence of the inflammatory reaction with granulocytes as first responders and macrophages arriving second [31]. As the pFN-dependent co-localization of macrophages with tumor cells was more sustained than co-localization with granulocytes, we decided to follow-up on the interaction of macrophages with pFN.

Tumor-associated macrophages are derived from circulating monocytes, which in response to specific stimuli, attach to the vascular endothelium, generate pseudopodia to extravasate, and then invade the perivascular tissue [4]. Paralleling these data, we demonstrate that the presence of pFN plays an active role in cell invasion by mediating the formation of an elongated macrophage phenotype after embedding monocytes in a 3D matrix of clotted plasma or fibrin–fibronectin. The elongation of macrophages is predictive for matrix-driven 3D cell invasion because the underlying change in cell shape in 3D can only take place if elongated cells infiltrate the surrounding matrix in a protease-dependent manner [32,33]. In addition, macrophage elongation and the underlying adhesive interactions with the extracellular matrix are a critical aspect of M2 polarization, suggesting that clotted plasma and pFN are specifically involved in the process of generating M2-polarized macrophages [22,34]. This concept is supported by data showing that macrophage elongation and M2 polarization can be achieved by culturing bone marrow-derived macrophages on fibronectin-coupled hydrogels [22]. Moreover, the M2 polarization of macrophages in clotted plasma containing endogenous pFN coincides with the expression of CD206 and CD163, two well-characterized markers of M2 polarization, while TNFα became downregulated [19]. This suggests that the presence of fibrin–fibronectin in the plasma clot that surrounds metastatic tumor cells serves the purpose of recruiting tumor-associated macrophages to the metastatic niche by facilitating extravasation and M2 polarization.

Inducing the elongated macrophage phenotype on fibronectin micropatterns represents a potent stimulus for M2 polarization that involves signaling through the actin cytoskeleton [22]. Cytoskeletal reorganization in macrophages can also be achieved by binding M-CSF to the receptor tyrosine kinase CSF1-R, which is known to induce an M2-polarized macrophage phenotype [35,36]. We confirmed the pro-adhesive effect of M-CSF in primary macrophages by demonstrating F-actin polymerization in the filopodia of elongated macrophages attached to cell culture plastic. The cell adhesion of macrophages was specifically activated by solubilized fibrin–fibronectin complexes but not by solubilized fibrin, pFN, or solubilized clotted plasma made from pFN-depleted plasma, suggesting that the complex formation of pFN with fibrin is essential for the pro-adhesive function of fibrin–fibronectin complexes. Soluble fibrin–fibronectin complexes have been shown to activate integrin αvβ3 on THP-1 macrophages, suggesting that integrin activation by fibrin–fibronectin is a function that macrophages have in common with tumor cells that invade clotted plasma in order to induce metastasis to the lungs [12,14]. In the present study, we show that the expression of integrin αvβ3 is necessary for macrophage elongation in fibrin–fibronectin clots, indicating that macrophage invasion in clots depends on the activation of integrin αvβ3. This could mean that the interaction of fibrin–fibronectin with integrin αvβ3 promotes macrophage extravasation during lung metastasis in a manner similar to tumor cell invasion [14]. This concept is in line with research showing that macrophage colonization in tumors is reduced in transgenic mice that fail to express integrin αvβ3 on myeloid cells [16]. Moreover, the fibrin–fibronectin-mediated ligation of integrin αvβ3 on macrophages could offset the pro-inflammatory effects of fibrin binding to macrophage integrin αMβ2 and therefore contribute to the supporting role of pFN in lung metastasis that we reported previously [14,27].

We recently showed that the presence of pFN causes upregulation of the receptor tyrosine kinase Tie2 in fibrin-embedded tumor cells and that knocking down Tie2 in these cells is associated with reduced invadopodia formation [13]. Paralleling these data, we found that elongation in clot-embedded macrophages depends on Tie2. Interestingly, this mechanism is intimately linked to pFN-mediated clot invasion, as Tie2 appears to be dispensable in kidney cancer and glioblastoma cells that invade fibrin spontaneously [12]. Kidney cancer and glioblastoma are known for the redundant activation of several receptor tyrosine kinases, whereas macrophages seem to specifically depend on Tie2, which has been shown to activate PI-3 kinase as well as PAK [37,38,39,40,41]. In addition, Tie2 has been shown to recruit and phosphorylate focal adhesion kinases in cooperation with integrin α5β1, a known fibronectin receptor [42]. Moreover, Tie2 undergoes crosstalk with integrin αvβ3, which enhances signaling in response to angiopoietin 2 in endothelial cells [43]. Tie2 was originally identified as an endothelial kinase but since then has been shown to be expressed in a subset of M2 macrophages with specific pro-angiogenic functions [10]. This is in line with the notion that M2 polarization is associated with proangiogenic functions [36]. Considering that M2 macrophages are able to invade clotted plasma while M1 macrophages stay largely round, our data suggest that blood clots contribute to the differentiation of pro-angiogenic effector cells during lung metastasis. Thus, further research in the interaction of macrophages with fibrin–fibronectin complexes is needed and could ultimately lead to the identification of treatment targets that inhibit the recruitment of regenerative macrophages to metastatic tumor cells.

## 5. Conclusions

We previously showed that fibrin–fibronectin promotes clot invasion in tumor cells in vitro and this mechanism correlates with a pro-metastatic effect of pFN in vivo [14]. Here, we demonstrate that the recruitment of macrophages to tumor cells is reduced in lungs from pFN-deficient mice. As circulating tumor cells are surrounded by blood clots in vivo, we established a requirement of pFN for macrophage elongation in clot-embedded macrophages in vitro. The elongation of macrophages in clotted plasma was associated with M2 polarization and depended on the expression of integrin β3 as well as Tie2. Together our data indicate that fibrin–fibronectin is an important modulator of macrophage interactions with clotted plasma. Moreover, they suggest that fibrin–fibronectin complexes contribute to the recruitment of myeloid cells to circulating tumor cells.

## Data Availability

The original contributions presented in this study are included in the article/Supplementary Materials. Further inquiries can be directed to the corresponding author.

## Supplementary Materials

The following supporting information can be downloaded at: https://www.mdpi.com/article/10.3390/cancers17233780/s1, Figure S1: M2 polarization of primary macrophages supports shape change and elongation in clotted plasma in vitro; Figure S2: M2 polarized macrophages do not make a fibronectin matrix in vitro; Figure S3: siRNA mediated knockdown of FN, Tie2 and integrin β3.
