# Supplementary material for: Plasma Fibronectin Drives Macrophage Elongation via Integrin β3–Tie2 Axis in Blood Clots
Source: Cancers (Basel). 2025 Nov 26;17(23):3780. doi: 10.3390/cancers17233780 (PMC12691047; doi:10.3390/cancers17233780)
Supplement: Supplementary file 1 [file cancers-17-03780-s001.zip › cancers-3952150-supplementary.pdf]

Supplementary Figure Legends

Supplementary Figure S1 *M2 polarization of primary macrophages supports shape change and elongation in clotted plasma in vitro.* Representative phase contrast images of plasma clot-embedded primary human macrophages 8 days after treatment with vehicle control (Untreated), macrophage colony stimulating factor (M-CSF), granulocyte-macrophage colony-stimulating factor (GM-CSF) or following the addition of interleukin 4 (IL-4) or the combined addition of lipopolysaccharide (LPS) and interferon-gamma (IFNγ) to the M-CSF treated cultures are shown. White arrows denote elongated macrophages. Scale bar, 100 μm.

Supplementary Figure S2 *M2 polarized macrophages do not make a fibronectin matrix in vitro.* Primary human macrophages were grown in the presence of macrophage colony stimulating factor on glass culture slides for 10 days to induce M2 polarization. Macrophages were then fixed with 4% paraformaldehyde, permeabilized with 0.2% triton x100, stained with anti-fibronectin (green, Millipore) or control IgG and analyzed by fluorescence microscopy. Actin was stained with phalloidin (red; Invitrogen) and nuclei were stained with mounting media containing DAPI (blue). Representative images are shown for FN (right micrograph) and FN overlayed with actin in the merged image (left micrograph). Scale bar, 50 μm.

Supplementary Figure S3 *siRNA mediated knockdown of FN, Tie2 and integrin β3.* (A), RT-PCR analysis of fibronectin (FN) mRNA expression in THP-1 cells 2 days after

transfection with siRNA against FN (siFN) compared to non-targeted control siRNA (siCTRL)(n = 2). (B), mRNA levels determined by RT-PCR in THP-1, 786-O, and U87MG cells to demonstrate knockdown of Tie2 (n = 2). Control siRNA was set to 1. Error bars show standard deviation. (C), western blotting for integrin β3 expression to demonstrate knockdown in THP-1 cells compared to non-targeted control siRNA. Ponceau S staining shows equal loading.

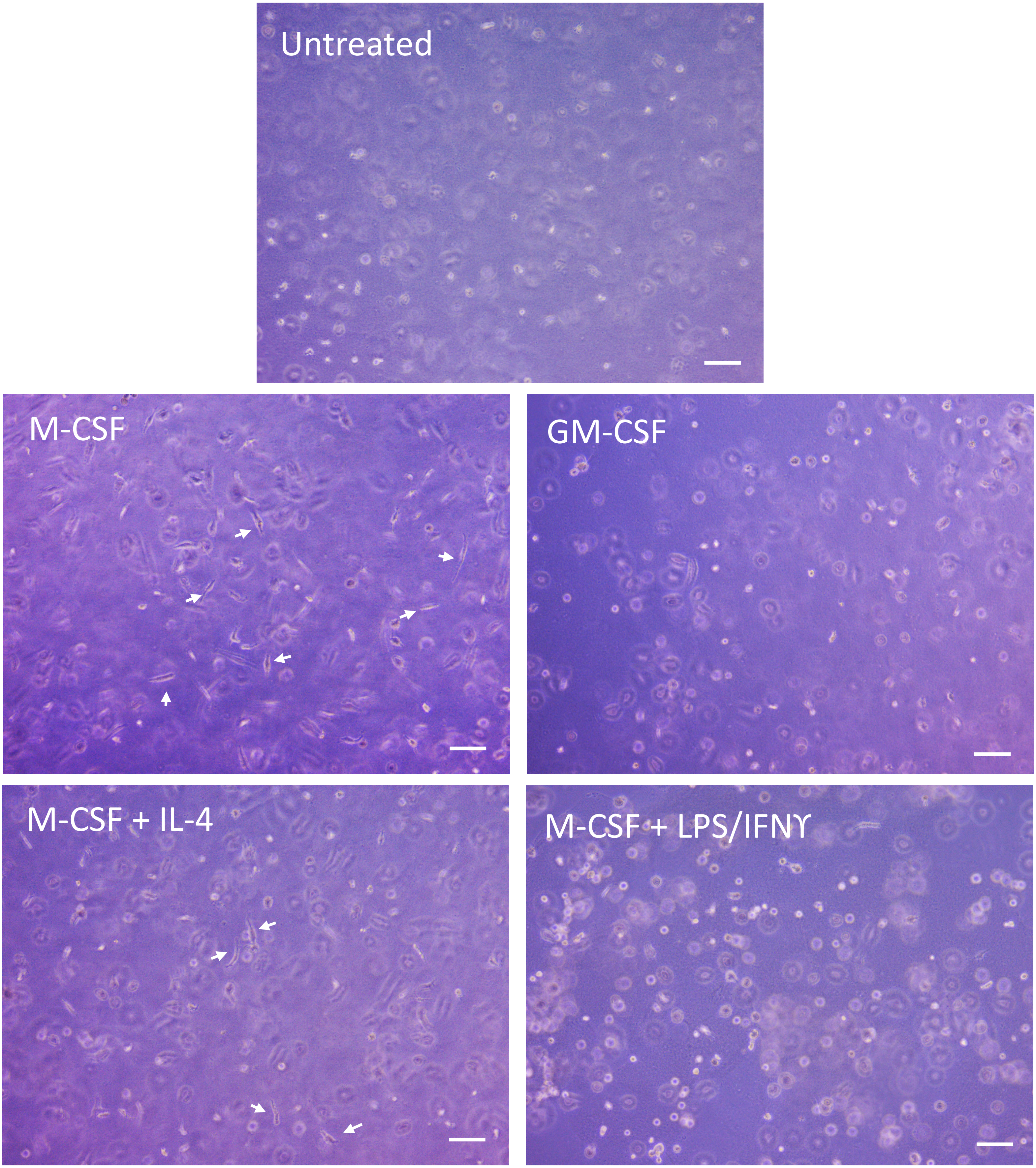

Knowles et al. Supplementary Figure S1

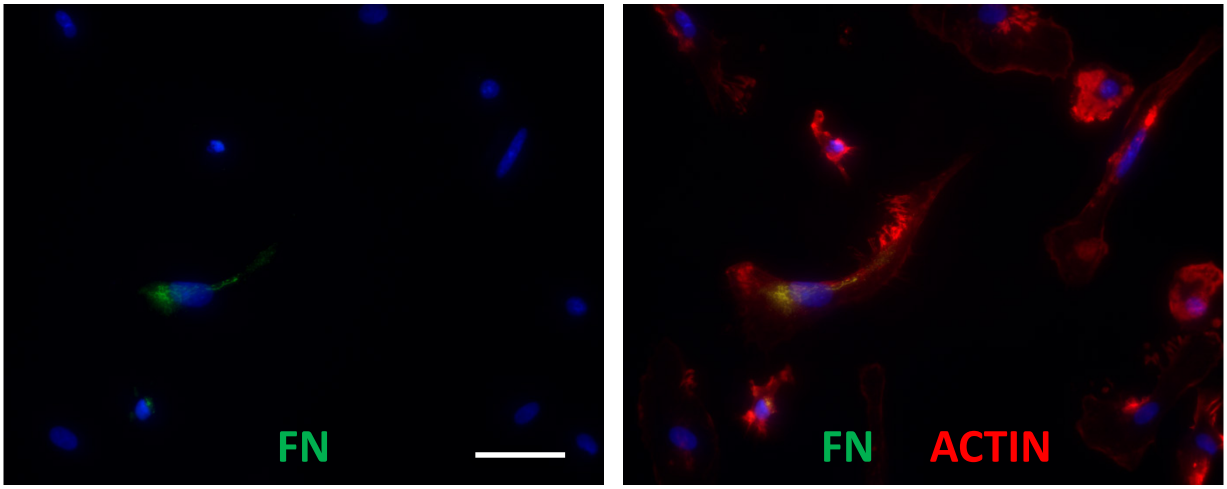

Knowles et al. Supplementary Figure S2

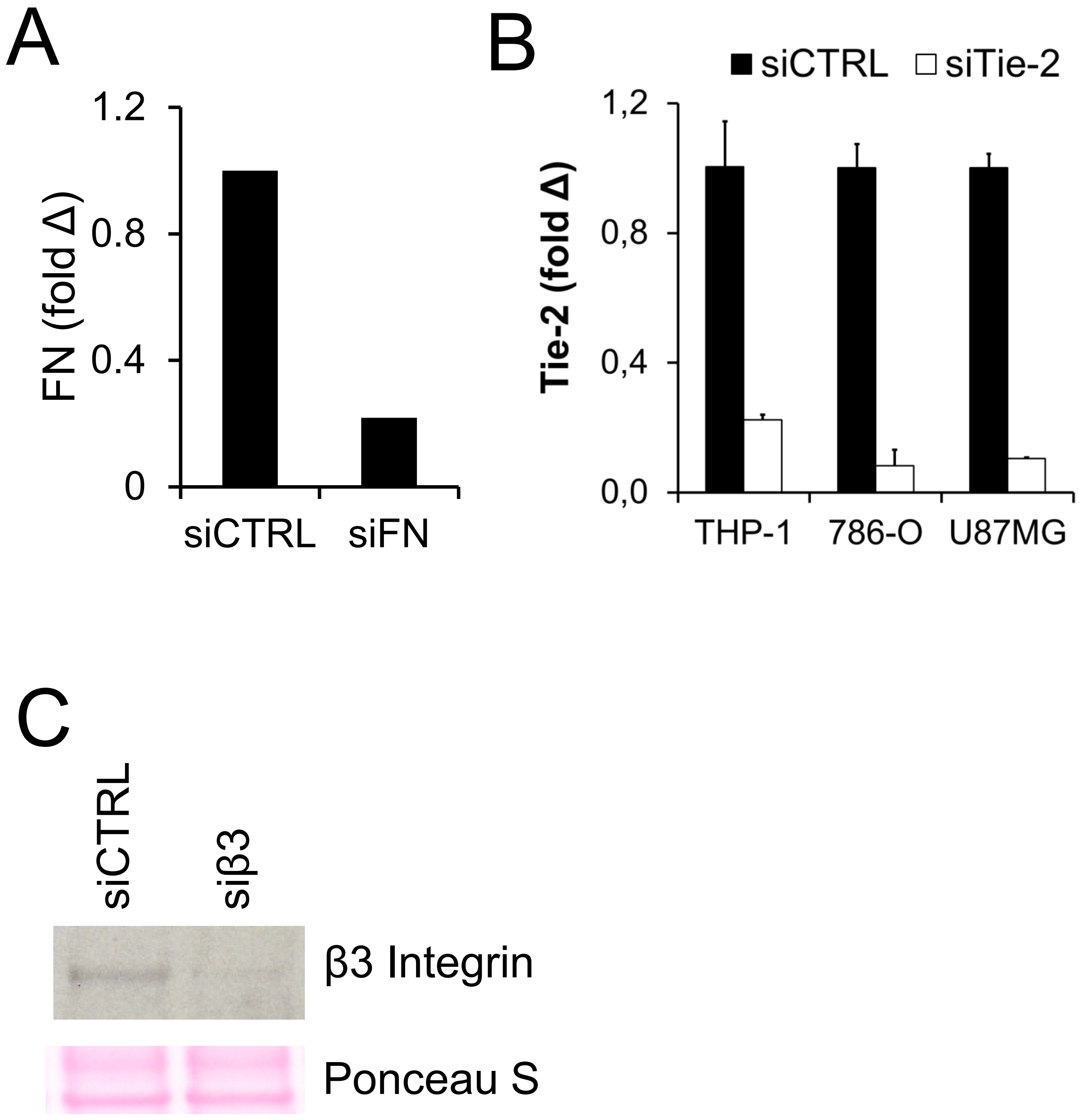

Knowles et al. Supplementary Figure S3
